# Statistical Analysis of Interactive Surgical Planning Using Shape Descriptors in Mandibular Reconstruction with Fibular Segments

**DOI:** 10.1371/journal.pone.0161524

**Published:** 2016-09-01

**Authors:** Megumi Nakao, Shimpei Aso, Yuichiro Imai, Nobuhiro Ueda, Toshihide Hatanaka, Mao Shiba, Tadaaki Kirita, Tetsuya Matsuda

**Affiliations:** 1 Graduate School of Informatics, Kyoto University, Kyoto, Japan; 2 Department of Oral and Maxillofacial Surgery, Rakuwakai Otowa Hospital, Kyoto, Japan; 3 Department of Oral and Maxillofacial Surgery, Nara Medical University, Nara, Japan; University of North Carolina at Chapel Hill, UNITED STATES

## Abstract

This study was performed to quantitatively analyze medical knowledge of, and experience with, decision-making in preoperative virtual planning of mandibular reconstruction. Three shape descriptors were designed to evaluate local differences between reconstructed mandibles and patients’ original mandibles. We targeted an asymmetrical, wide range of cutting areas including the mandibular sidepiece, and defined a unique three-dimensional coordinate system for each mandibular image. The generalized algorithms for computing the shape descriptors were integrated into interactive planning software, where the user can refine the preoperative plan using the spatial map of the local shape distance as a visual guide. A retrospective study was conducted with two oral surgeons and two dental technicians using the developed software. The obtained 120 reconstruction plans show that the participants preferred a moderate shape distance rather than optimization to the smallest. We observed that a visually plausible shape could be obtained when considering specific anatomical features (e.g., mental foramen. mandibular midline). The proposed descriptors can be used to multilaterally evaluate reconstruction plans and systematically learn surgical procedures.

## Introduction

Vascularized fibular transfer has become a common surgical procedure in mandibular reconstruction [1, 2]. During this surgical procedure, fibular segments are transplanted into the mandibular defect with consideration of the mandible’s curved shape and mastication functions. The advantages of using native bone compared with artificial bone transplantation are quick engraftment and earlier formation of new bone. However, during preoperative planning, it is necessary to determine the optimal characteristics of the osteotomy for fibular shaping, the number of partitions of the fibula, and the proper placement of the fibular segments in the mandible. The use of a patient’s original fibular segments presents a more complex planning problem than does the use of artificial bones. Even with clinical efforts using three-dimensional (3D) computed tomography (CT) images, additional cutting during the operation is often needed. Therefore, accurate fibular osteotomy and mandible reconstruction remain challenging, and are still dependent on the surgeon’s experience and technical skills.

In recent years, virtual surgical planning has gained attention in terms of supporting the preoperative design of mandibular reconstruction using patients’ CT volume data [3–8]. Such computer-aided planning permits the rapid design of a patient-specific surgical procedure as well as visualization of 3D structures that can be shared among surgeons, technicians, and other medical staff members [7, 8]. Various reconstruction plans can be considered using the rendered image of the patient’s own fibula superimposed onto the mandibular defect. Another advantage is that the results of these planning activities are directly available for stereolithographic model generation using 3D printer systems [9, 10]. With the planning data, fibular cutting guides and a plate-bending template can be manufactured to aid in further reconstruction planning [11–14].

Preoperative optimization of the reconstructive procedure is essential to improving surgical outcomes while reducing intraoperative burdens. Several recent clinical studies have investigated the accuracy of virtual planning by comparing the plan and the reconstruction results before and after the surgery [15–18]. Fibular segments, prefabricated surgical plates, and cutting guides planned with software were compared with actual treatment results. To optimize the patient-specific procedure, the virtual plan should be quantitatively assessed as completely and accurately as possible during the preoperative planning stage. However, conventional planning procedures are mostly based on manual operation and require a trial-and-error process to refine the reconstruction plan [7, 10]. Biomechanical behavior of the mandible after marginal resection has been investigated based on finite element analysis [19]. For mandibular reconstruction, the postoperative facial appearance is a key factor to maintain patients’ quality of life. Although a mirror image of the unaffected mandible onto the mandibular defect has been developed to ensure symmetrical reconstruction [20, 21], this approach does not evaluate the difference between pre- and post-operative facial appearance. From an aesthetic point of view, restoration of the patient’s native mandible is essential in fibular transfer surgery; however, quantitative analysis of the personalized reconstruction procedure has not been well investigated.

We focused on designing interactive planning software that allows the user to create a reconstruction plan of fibular transplantation while quantitatively comparing the plan with the patient’s original mandible. Because coordination and placement of fibular segments requires complex procedures including 3D translation and rotation in the virtual space, it is often difficult to achieve fine adjustment of the transfer plans, such as bone-to-bone contact. For this reason, the use of virtual planning is currently limited to biomedical engineers, and requires additional communication between surgeons and engineers. To the best of our knowledge, our preliminary work is the only research on the use of a virtual planning system that allows quantitative shape analysis on the pre- and post-operative mandibular arc for fibular transfer surgery [22, 23]. Further statistical analysis of the surgical planning data and investigation of implicit factors affecting the decision-making process are important for the computer-assisted surgery community and for designing next-generation planning software.

In this paper, we present an improved preoperative planning design with quantitative shape indicators for fibular transfer in mandibular reconstruction and report the findings obtained through statistical analysis of the interactive planning results. We focus on aesthetic reconstruction of the postoperative mandibular arc, and newly introduce the *shape distance* to quantify the local difference between the planned mandibular reconstruction and patient’s native mandible. The user can refine the preoperative plan using the 3D map of the shape distance as a visual guide. We also conducted a retrospective user study with oral surgeons and dental technicians and obtained 120 reconstruction plans using 3D CT images. The obtained results and possible factors affecting the decision-making process are statistically discussed in the latter part of the paper.

## Methods

### Fibular Transfer Planning

The use of the medical images has been approved by Nara Medical University’s Ethics Committee. The images are stored in Nara Medical University. Yuichiro Imai and Nobuhiro Ueda can access the data and personal information. The experiments were conducted after anonymization of the patient information.

**Fig 1:**
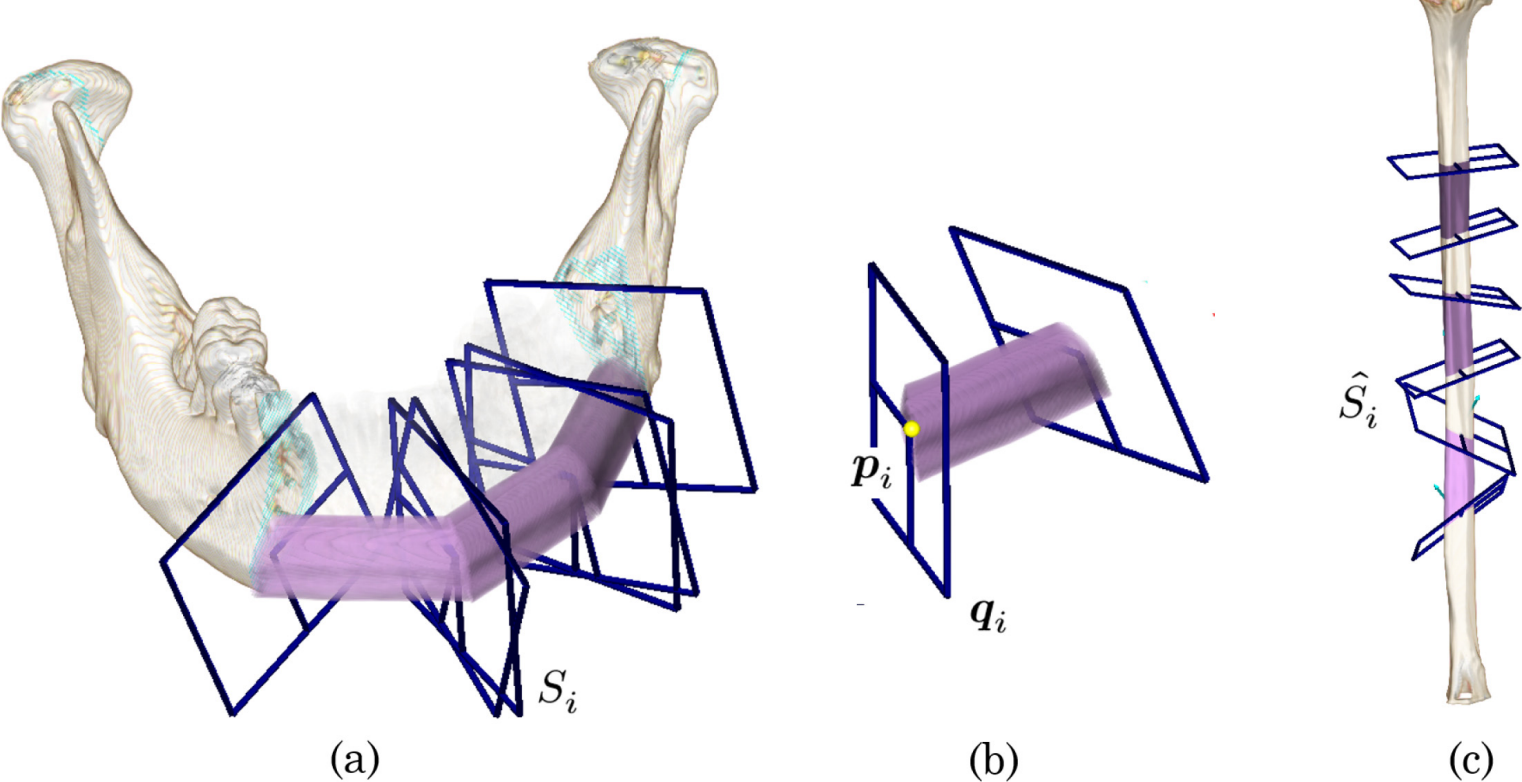
Geometrical description of the fibular transfer simulation. (a) Three fibular segments superimposed on the resection area. (b) Native fibular volume annotated with the surgical plan

The resection area on the mandible is first planned while considering the most appropriate margins for removal of cancerous lesions or reconstruction of mandibular defects. The resection area is determined using the boundary surfaces in the volumetric mandibular image *I* as shown in Fig 1A. Mandibular reconstruction using fibular segments can be efficiently simulated using connection points between the fibular segments and corresponding virtual planes [22, 23]. In this paper, we introduce a set of 2*N* reconstruction parameters ***θ*** = [***θ***_0_,***θ***_1_,…,***θ***_2*N*−1_] to formulate fibular transfer planning with *N* fibular segments, where the reconstruction parameter ***θ***_*i*_ represents the position and the orientation of the boundary surface. For the *N* fibular segments, 2*N* controllable planes *S*_*i*_ (*i* = 0,1,…,2*N*−1) are used to define the resection area and osteotomy lines for fibular shaping. Here, ***p***_0_ and ***p***_2*N*−1_ on the virtual planes represent the 3D positions of the connection points between the patient’s native mandible and the fibular segments. The other points ***p***_*i*_ (*i* = 1,…,2*N*−2) are used as connection points between two fibular segments. Three-dimensional rotation of the plane *S*_*i*_ is described using a 4D vector ***q***_*i*_; *i*.*e*., quaternion. A set of the connection point ***p***_*i*_ and the quaternion ***q***_*i*_ give a simple form for the boundary surface (Fig 1B). Based on this formulation, a reconstruction parameter ***θ***_*i*_ is described as follows:

$$\theta_{i}=[p_{i},q_{i}]$$ (1)

The corresponding virtual planes ${\hat{S}}_{i}$ (*i* = 0,…,2*N*−1) are placed on the fibular image *I*_*f*_ to define the fibular segments. Fig 1C shows three fibular segments defined by six planes: ${\hat{S}}_{0}$, … ${\hat{S}}_{5}$ (termed a three-segment procedure in this paper). A two-segment procedure is similarly defined using four virtual planes. Using the reconstruction parameter ***θ***_*i*_, each plane ${\hat{S}}_{i}$ is mapped to the corresponding plane *S*_*i*_ placed on the resection area of the mandible. The connection points between two fibular segments are linked and constrained in the same position. The normal vectors of the virtual planes between two fibular segments are constrained in the same direction. For example, when translating or rotating the virtual surface *S*_1_ between the left and central segments, the virtual surface *S*_2_ is simultaneously updated and the corresponding virtual surfaces ${\hat{S}}_{1}$ and ${\hat{S}}_{2}$ are also updated while maintaining the distance and the relative angle. Fig 1A shows the simulation results with the oriented fibular image *I*′_*f*_ that is segmented and transformed by the reconstruction parameter set ***θ*** from the fibular CT image *I*_*f*_. Thus, a reconstruction plan is uniquely defined by ***θ***. This scheme also enables the user to refine the plan while observing simulation results on both mandibular reconstruction and fibular osteotomy.

**Fig 2:**
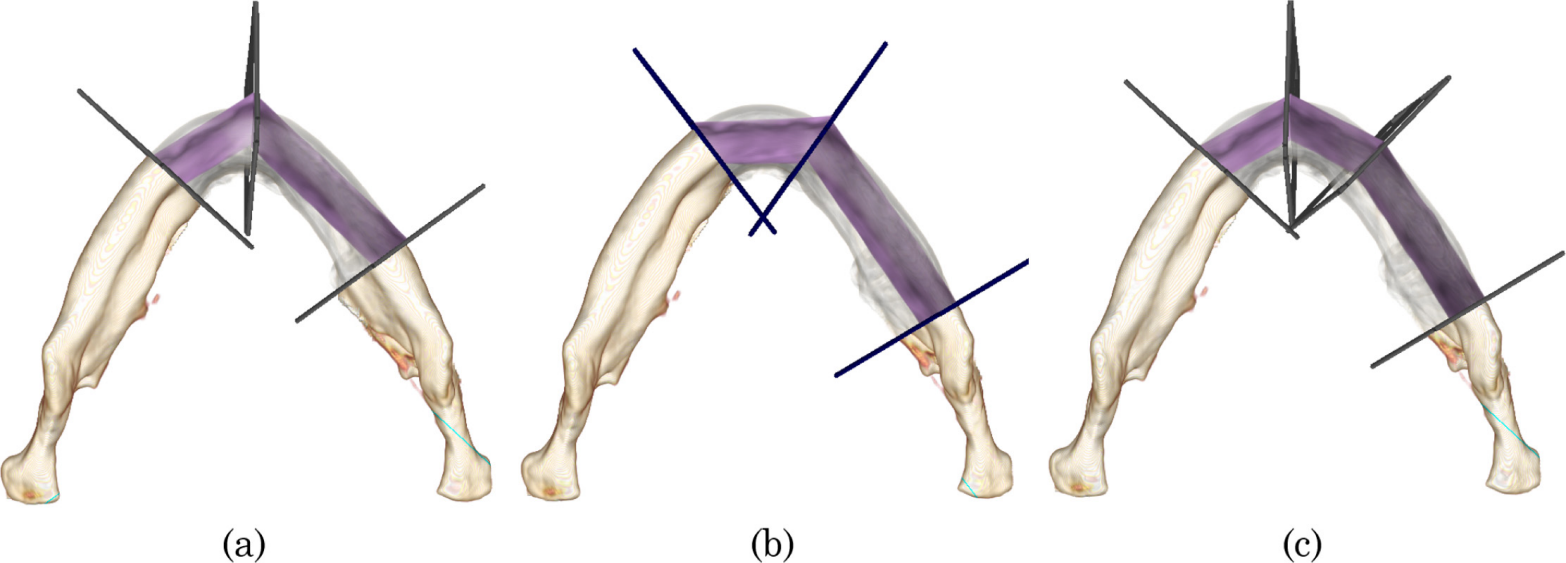
Examples of fibular transfer simulation. (a, b) Two-segment procedures with different placement of the connection points. (c) Another three-segment procedure

Fig 2 shows three possible examples of mandibular reconstruction suggested by surgeons. Fig 2A and 2B shows two-segment procedures with two different placements of the connection point ***p***_1_. This variation comes from the differences in the geometrical features of the resection area, the patient’s native mandible, and the fibular segments. This decision has been empirically made based on the estimated postoperative visual appearance, and it is still quantitatively unclear which placement leads to better reconstruction. Fig 2C shows the three-segment procedure using four virtual planes. Notably, the placement of the connection point differs from that in Fig 1A. From a geometrical viewpoint, the three-segment procedure probably reconstructs the curved shape of the native mandible better than does the two-segment procedure. In spite of this, the two-segment procedure is still a good choice for actual surgery because of the higher intraoperative burden and the required surgery time associated with the three-segment procedure [23]. Here, given the patient’s CT images and the specific resection area on the mandible, we meet the following two fundamental questions: How many fibular segments should be chosen and where should the connection points be placed? Our motivation for this study was the creation of a systematic approach to these questions, and we targeted quantitative analysis of the reconstruction plans to explore planning factors and further automation of the planning procedures. In the next section, we introduce some shape descriptors to quantify the reconstruction plans.

### Shape Descriptors

**Fig 3:**
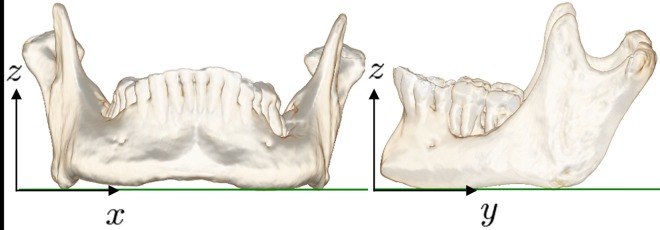
Mandibular tangent plane uniquely defined on the mandible image.

The posture of the patient during measurement affects the relative position and orientation of the mandible in the 3D CT images. For quantitative evaluation of the reconstruction plans, a unique 3D coordinate system should be determined for the individual mandibular image *I*. In general, the surgeons carefully consider reconstruction on the shape of the chin and lower edge of the patient’s native mandible. Based on this observation, a *mandibular tangent plane* that is uniquely defined for the 3D surface of the mandible is created. Fig 3 shows an example of the *mandibular tangent plane* placed at the mandibular lower edge. This reference plane can be computed as a ground plane when a rigid object drops to the ground and reaches a stable state. In this paper, the normal vector of the mandibular tangent plane is referred to as the *z* direction.

Shape differences between the patient’s native mandible *I* and the implanted fibular segments *I*′_*f*_ are the main factors that affect the postoperative appearance of the mandible. We herein introduce the *shape distance E*_*s*_ as a shape descriptor of the reconstruction plan ***θ*** described as follows:

$$E_{s}=\frac{1}{n}\sum_{j=0}^{n-1}d(x_{j},X_{j})$$ (2)

where ***x***_*j*_ denotes a 3D sampling point on the implicit surface of the mandibular image *I*, and ***X***_*j*_ is the corresponding sampling point on that of the fused fibular image *I*′_*f*_. *d* is the Euclidean distance between the given two points, and *n* is the number of sampling points.

**Fig 4:**
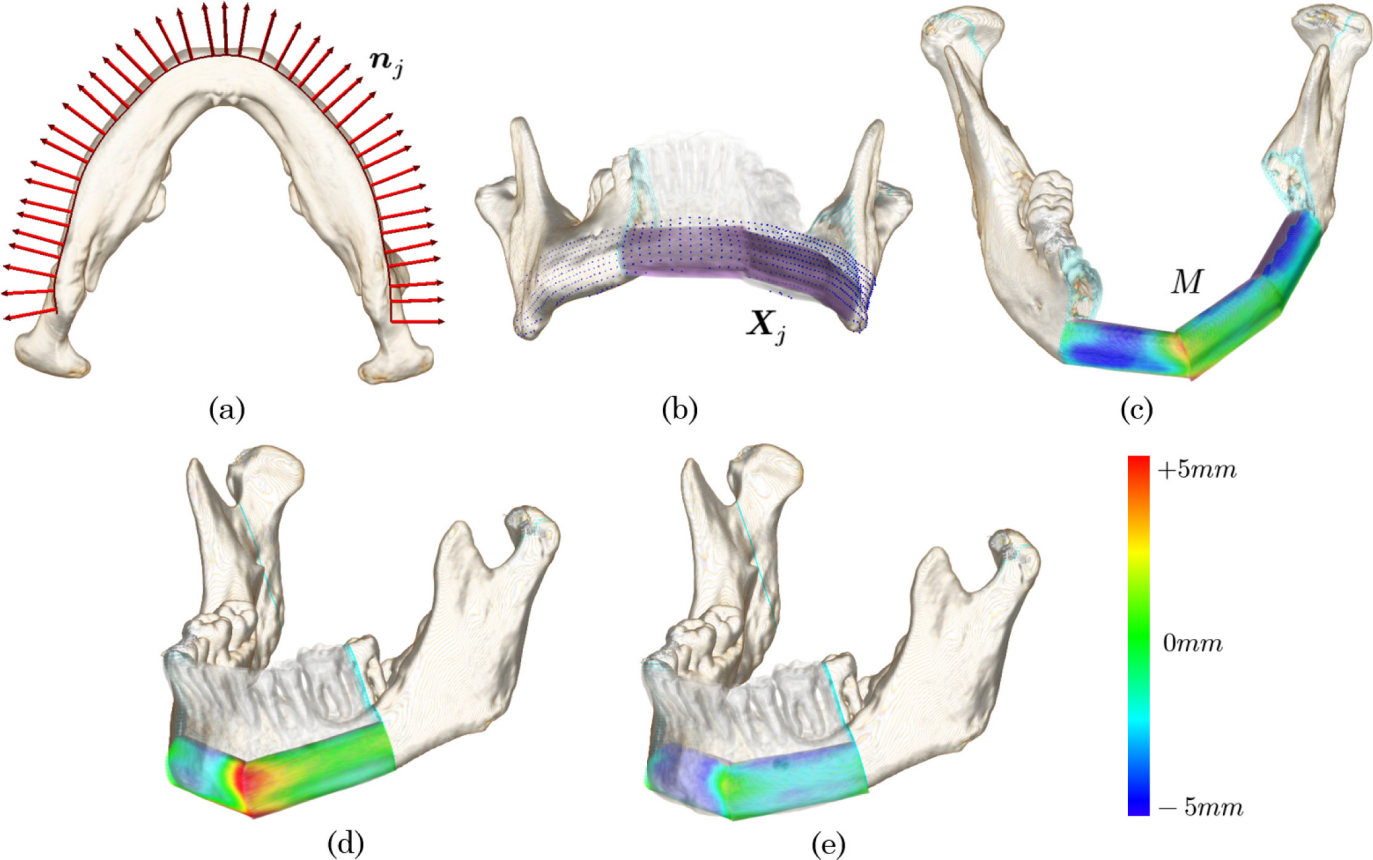
Shape distance computation and mapping on the oriented fibular segments. (a) Curved line detection of the mandible. (b) Detection of a set of the sampling points (***x***_*j*_, ***X***_*j*_). (c) Spatial distribution of the *d*(***x***_*j*_,***X***_*j*_) overlaid on the fibular image forms the shape distance map *M*. (d, e) The shape distance map changes by replacement of the connection point

The distribution of *d*(***x***_*j*_,***X***_*j*_) for all sampling points on the fibular segments forms a *shape distance map M*. Fig 4 shows an example of the *shape distance map M* and the outline of the computation algorithm. The computation algorithm is described by the following four steps:

**Step 1** The curved lines on the mandibular surface are first obtained by scanning slice images of the mandibular volume *I*. The slice images are generated parallel to the mandibular tangent plane.
**Step 2** The curved lines are re-sampled with a certain voxel distance *Δ****x***, and the sampling points ***x***_*j*_ and the corresponding normal vectors ***n***_*j*_ are obtained (Fig 4A).
**Step 3** The corresponding sampling point ***X***_*j*_ of ***x***_*j*_ is detected by scanning the oriented fibular image *I*′_*f*_ from ***x***_*j*_ in the direction of ***n***_*j*_ (Fig 4B).
**Step 4** *d*(***x***_*j*_,***X***_*j*_) is computed for all sampling points ***x***_*j*_ and mapped to the fibular segments in the rendering pipeline (Fig 4C).

The *shape distance map M* quantifies the local shape distance of the two implicit surfaces of *I* and *I*′_*f*_. The red color overlaid on the fibular segments in Fig 4D estimates that the part of the fibular implants near the connection point protrudes from the patient’s native mandible. In contrast, the blue color indicates depression from the patient’s native mandible (Fig 4E). The green color indicates that the local distance is equal to zero; that is, the placed fibular implants locally fit the patient’s native mandible. The color distribution changes in response to replacement or rotation of the fibular segments. Because lack of depth information or occlusion of the volumetrically rendered image can cause misalignment of the fibular segments, heat map representation of the local shape distance can serve as intuitive visual guidance to refine the reconstruction plan. Notably, the contour error introduced in recent work [23] was limited to symmetrical lesions of the central part of the mandible. The herein-proposed *shape distance* supports curved surfaces and asymmetrical lesions including the lateral region of the mandible and provides more a generalized measure with which to evaluate the local difference between the reconstructed mandible and the patient’s original mandible.

The *shape distance E*_*s*_ can be used to evaluate the average shape differences between curved surfaces, but it cannot be used to evaluate local difference such as the degree to which fibular segments extend beyond the resection area. Although the protruding part of the fibular implants could be additionally resected during the surgery, the available margin for resection is restricted within the thickness of the cortex. Additionally, the reconstructed mandible must have sufficient volume to maintain its postoperative mastication function and physical strength as a part of the facial bone structure. For multilateral evaluation of the planning procedures, the maximum projection *E*_*p*_ and the minimum length of the fibular segments *E*_*l*_ defined in Eq 3 and Eq 4, respectively, are also taken into account.

$$E_{p}=\max_{j\in \{0,\ldots ,n-1\}}n_{j}(x_{j}-X_{j})$$ (3)

$$E_{l}=\min_{j\in \{1,\ldots ,2N-1\}}d(p_{i},p_{i-1})$$ (4)

### Planning software and interface

The overall algorithms for visualization and computation of the shape descriptors were implemented using C++, GLSL, and CUDA and integrated into interactive planning software for mandibular reconstruction with fibular segments. The free software version biGAKU is available from our website (http://www.bigakuapp.com/en/about/), and the supplemental movies (S1 Movie) provide a demonstration. In the developed system, all operations for preoperative planning can be performed using a 2D pointing device such as a standard mouse or a touch interface. The user can interactively manipulate the parameter ***θ***_*i*_ of the placed virtual planes (*e*.*g*., translation and rotation) to refine the overall reconstruction planes. In response to the user’s operation in one virtual plane, other virtual planes are updated simultaneously to maintain their connection and geometrical constraints. Parallel computation on a graphical processing unit allows for updating of the *shape distance map* at an average rate of more than 20 frames per second. A set of the local shape distance *d*(***x***_*j*_,***X***_*j*_) is spatially interpolated and mapped with the heat map color on the fibular segments.

## Experiments and Results

The experiments were designed to quantitatively analyze medical knowledge of and experience with decision-making in preoperative planning of mandibular reconstruction. As described in Section 2, we specifically focused on the number of fibular segments and the placement of their connection points. Although the conventional work limitedly investigated symmetrical reconstruction of the central part of the mandible [23], the present study used the developed software biGAKU and targeted asymmetrical cases with a wide range of cutting areas, including the mandibular sidepiece. Four medical staff members (one oral surgeon and two dental technicians of Nara Medical University and one oral surgeon of Otowa Hospital) participated in our experiments.

### Data Preparation

Ten CT datasets obtained from both the head and foot were applied to the developed software. The data were selected from actual patient cases except for Case 6. Case 6 was a healthy mandible that represented the typical mandibular shape of an adult. The CT slice images were first re-sampled as regularized volume data with 256^3^ voxels. The mandible region *I* was extracted from the head CT data, and the fibular image *I*_*f*_ was extracted from the foot CT data. This segmentation step was completed in 1 to 3 minutes for each case based on selection of the region of interest and manual removal of voxels with a 3D cutting tool [24]. To retrospectively reproduce the preoperative planning while maintaining reproductivity, we defined five cutting planes based on the anatomical feature points of the mandible: *C*_*k*_ (*k* = 0, …, 4). The rotations of the cutting planes are uniquely determined because they are perpendicular to the outlines of the mandibular contour.

- *C*_0_: mandibular ramus
- *C*_1_: midpoint of *C*_0_ and *C*_4_
- *C*_2_: midpoint of chin and *C*_4_
- *C*_3_: midpoint of *C*_2_ and *C*_4_
- *C*_4_: mental foramen

**Fig 5:**
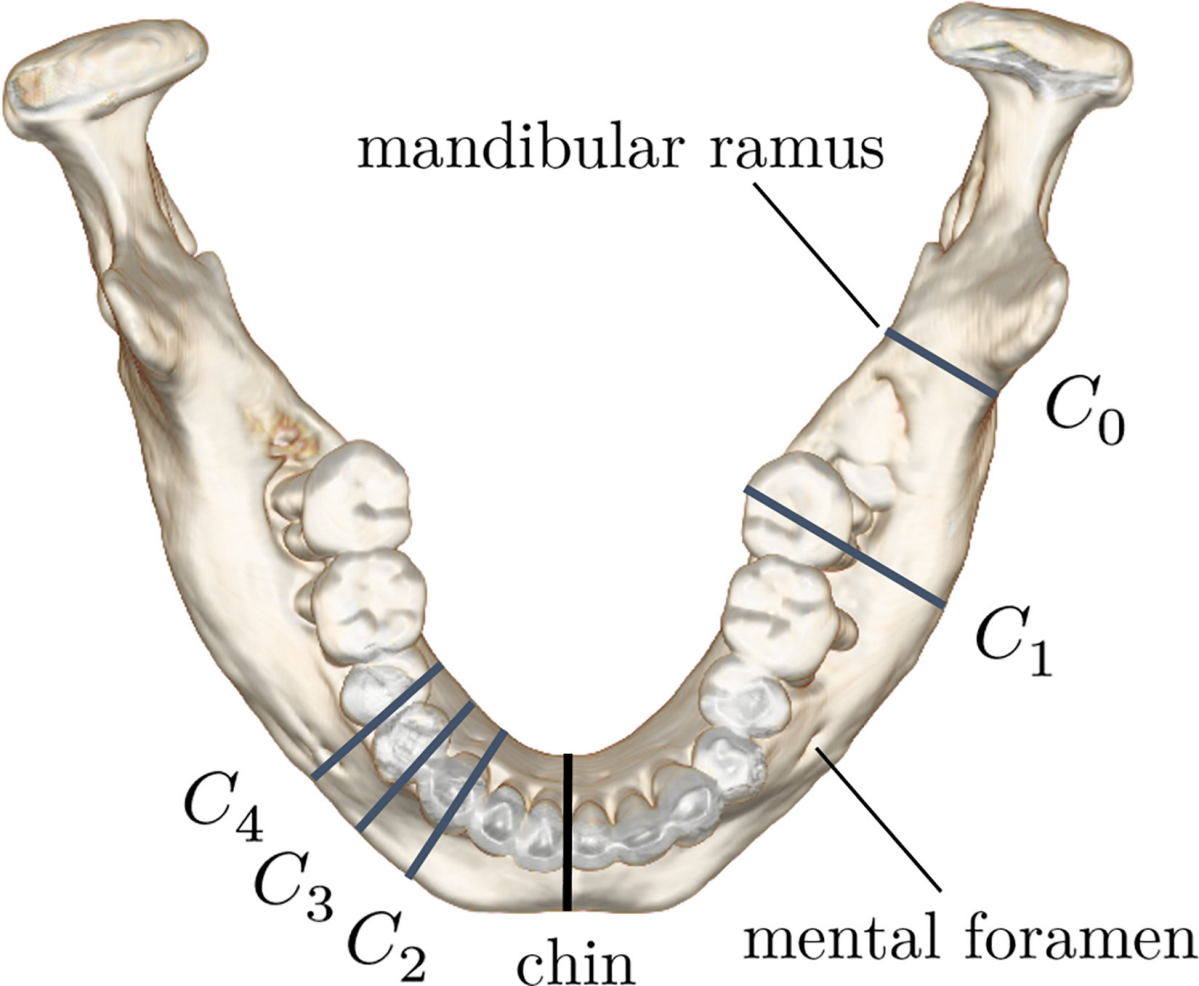
Five cutting planes based on anatomical features of the mandible. *C*_0_ was placed on mandibular ramus and *C*_4_ was placed on the mental foramen. *C*_1_, *C*_2_, and *C*_3_ were uniquely defined by *C*_0_ and *C*_4_.

Six patterns of the asymmetric cutting region [(*C*_0_,*C*_2_), (*C*_0_,*C*_3_), (*C*_0_,*C*_4_), (*C*_1_,*C*_2_), (*C*_1_,*C*_3_), and (*C*_1_,*C*_4_)] were then defined from a combination of the cutting planes (see Fig 5). We confirmed that these cutting patterns are similar to those used in past surgeries.

### Planning Procedures

In the experiment, the surgeons and technicians (hereafter termed participants) reproduced the surgical planes based on visualization of the patient’s mandible reconstructed with fibular segments. The virtual planes were operated by experimenters familiar with system operation; this allowed the participants to concentrate on the decision-making process for the planning procedure and reduced the required time and variation in the planning operation. During this experiment, two types of mandibular reconstruction using two or three fibular segments were interactively simulated while displaying the current states in two monitors with the same specification (*i*.*e*., same window size and same resolution). The participants watched both displays and determined the most suitable position of the connection points and angles of the virtual surfaces. When all participants agreed with the planning results, the reconstruction plan was fixed and recorded.

During the planning experiments, 60 resection area patterns (10 CT datasets and 6 types of resection areas) were presented in a random order to avoid bias from the order of effects, and 120 planning results on 2- and 3-segment procedures were obtained. The average shape distance *E*_*s*_, the maximum projection *E*_*p*_, and the minimum length of the fibular segments *E*_*l*_ were recorded for each planning procedure. After the two reconstruction plans with two or three segments were decided for each resection area, the experimenter requested that the participants determine which plan is empirically suitable and to answer positive or negative regarding their confidence in the decision. As this experiment aimed to determine the best plan for 60 resection areas, participants were not allowed to accept both plans. The four participants discussed this among one another and provided a single answer regarding the most suitable number of segments and the confidence in their decision.

## Results

Table 1 shows a set of the number of fibular segments that were selected by the participants for 60 resection area patterns. The brightness of the color indicates the degree of confidence; that is, a dark color means that the participants were confident in their decision, and a light color indicates negative confidence in the selection of the number of segments. The color distribution shows interesting results regarding the quantification of the surgeons’ decision making for the reconstruction plan. (*C*_1_,*C*_2_), (*C*_1_,*C*_3_), and (*C*_1_,*C*_4_) are the relatively smaller resection areas, and a two-segment procedure was chosen for these areas. The participants’ confidence shows that the decision became more difficult as the resection area increased in size. In contrast, (*C*_0_,*C*_4_) is the largest resection area, and a three-segment procedure was selected. The number of fibular segments selected for (*C*_0_,*C*_2_) and (*C*_0_,*C*_3_) differed between Cases 1 and 3 despite the fact that their resection areas were configured as anatomically similar. Therefore, we focused on the difference and further confirmed the details of the planning results.

**Table 1:** Number of fibular segments selected for 60 resection areas and the confidence in the decision. Six patterns of the asymmetric cutting region were defined using five cutting planes. A dark color indicates that the participants were confident, and a light color indicates negative confidence in the decision regarding the number of segments.

	case 1	case 2	case 3	case 4	case 5	case 6	case 7	case 8	case 9	case 10
**(*C***_**0**_**, *C***_**2**_**)**	2	2	3	2	2	2	2	2	3	2
**(*C***_**0**_**, *C***_**3**_**)**	2	2	3	2	2	2	2	2	2	2
**(*C***_**0**_**, *C***_**4**_**)**	3	3	3	3	3	3	3	3	3	3
**(*C***_**1**_**, *C***_**2**_**)**	2	2	2	2	2	2	2	2	2	2
**(*C***_**1**_**, *C***_**3**_**)**	2	2	2	2	2	2	2	2	2	2
**(*C***_**1**_**, *C***_**4**_**)**	2	2	2	2	2	2	2	2	2	2

**Fig 6:**
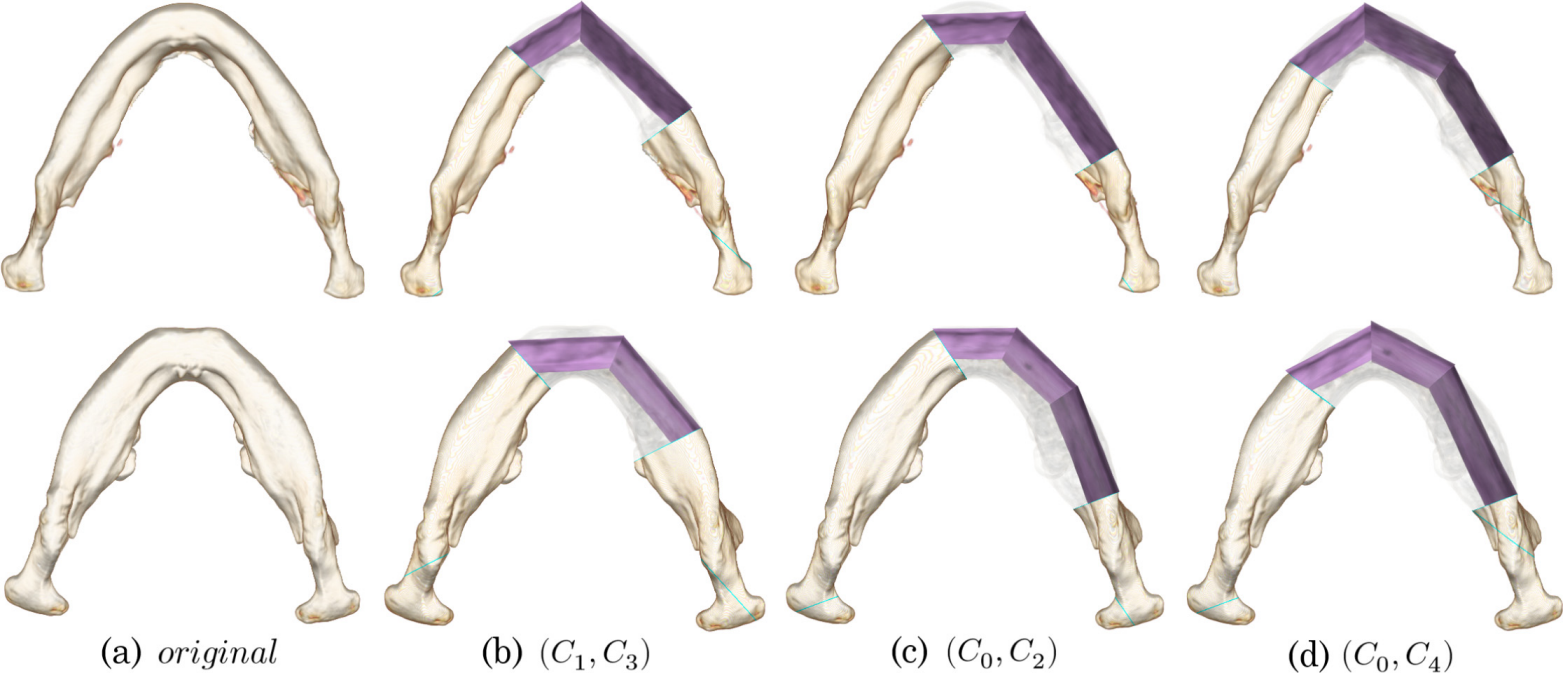
**Characteristic planning examples of Cases 1 and 3 for the resection areas (*C***_**1**_**,*C***_**3**_**), (*C***_**0**_**,*C***_**2**_**), and (*C***_**0**_**,*C***_**4**_**).** The number of fibular segments and the location of the connection points can vary in spite of the anatomically similar resection areas.

The upper images in Fig 6 show the planning results for Case 1, and the lower images show the planning results for Case 3. Fig 6A is the original CT image, and Fig 6B–6D shows the planning examples for the resection areas (*C*_1_,*C*_3_), (*C*_0_,*C*_2_), and (*C*_0_,*C*_4_), respectively. The original CT images confirm that the mandibular thickness of Case 1 is small and the curvature around the midline of the face is large, which can be represented as a *v*-shaped mandible. In contrast to Case 1, Case 3 can be represented as a *u*-shaped mandible, which has thicker bone and a smaller curvature around the midline. Regarding the reconstruction plans in Fig 6B, two fibular segments were chosen for both cases, but the connection point was differently placed at the midline for Case 1 and around the mental foramen for Case 3. This suggests that the participants change the priority of the reconstruction point by considering the shape of the mandible. The reconstruction plans in Fig 6C show that a two-segment procedure was selected for Case 1 and a three-segment procedure was selected for Case 3. Additionally, the connection points placed around the mental foramen slightly protrude from the native surface of the patient’s mandible. As shown by the reconstruction plans in Fig 6D, three fibular segments were used for both cases. One connection point was placed around the midline, and the other connection point was placed near the mental foramen. Specifically for Case 3, the local shape distance would be small if the right connection point was placed further to the right. However, this planning result suggests that the participants would perform much of the reconstruction around the mental foramen rather than minimize the local shape difference.

**Fig 7:**
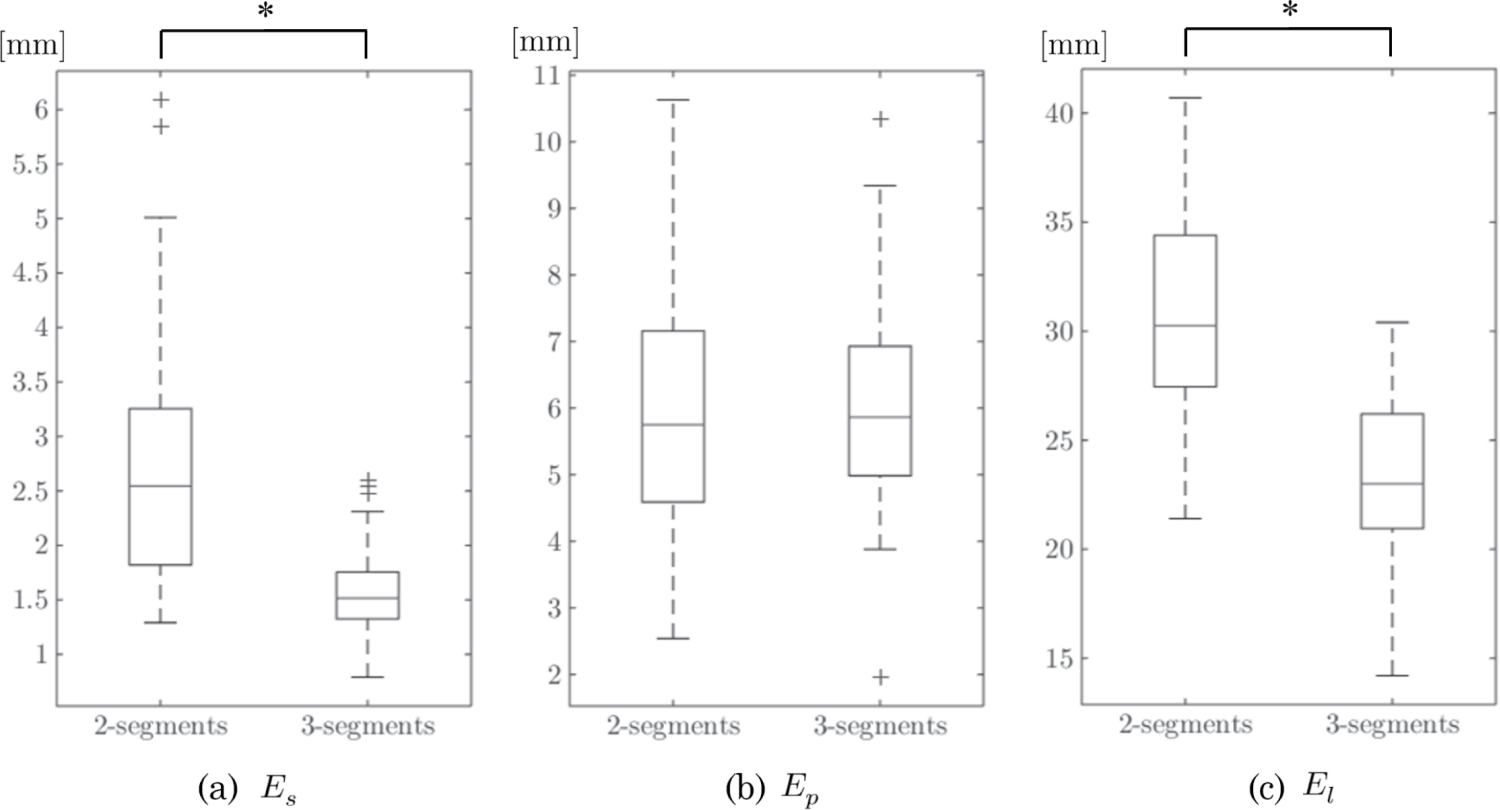
Statistical comparison of the three indicators between two- and three-segment procedures. The first and third quartile points are expressed using the box, and the median values are expressed using lines inside the box. One-way analysis of variance (ANOVA) was applied to test the obtained values of the shape indicators.

**Fig 8:**
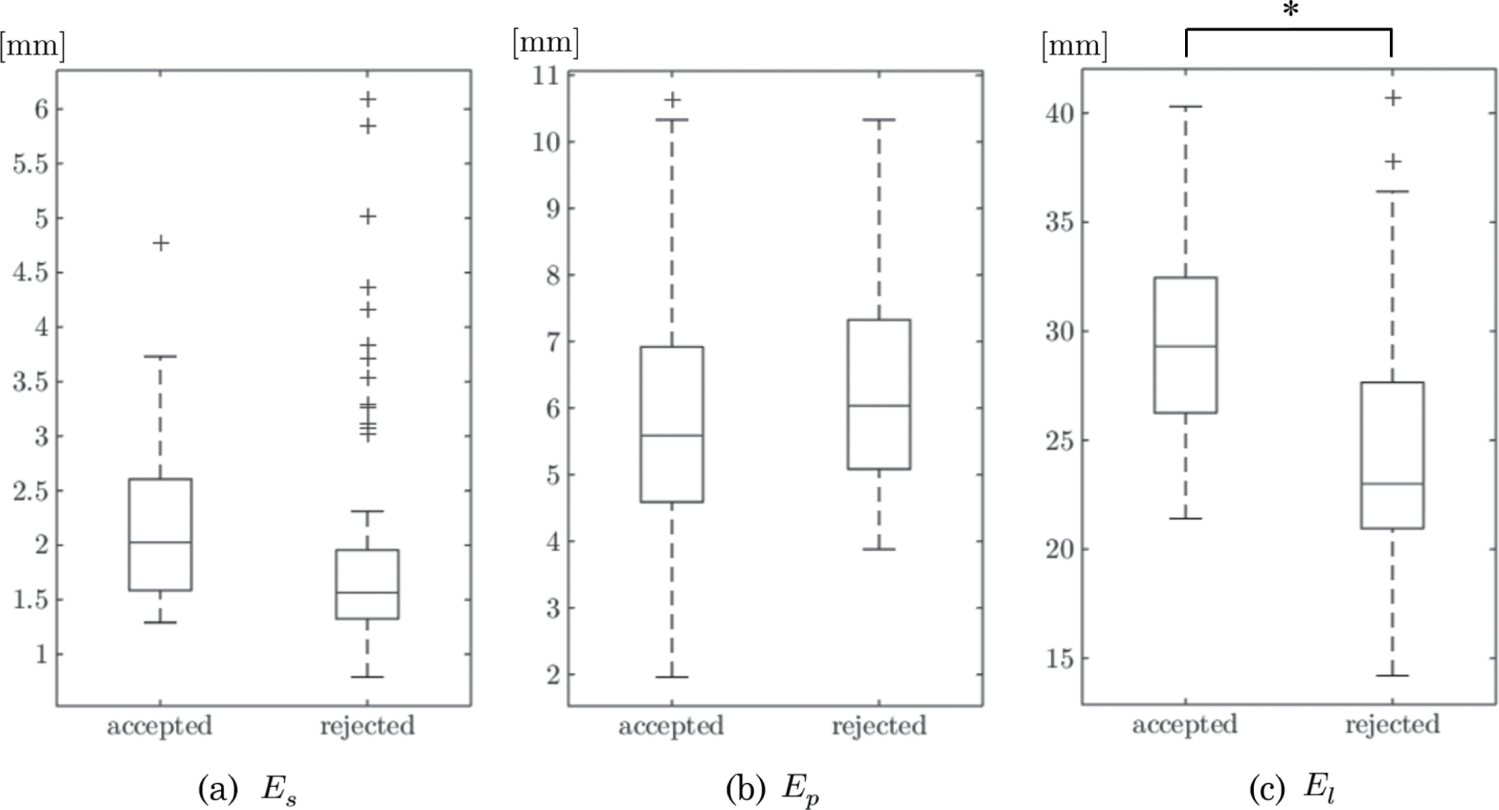
Statistical comparison of the three indicators between the accepted and rejected procedures. The box plots indicate the valuable findings of the surgeons’ decision making on fibular transfer surgery.

Table 2 summarizes the averages and standard deviations of the 3 proposed indicators computed from the 120 obtained planning results. The box plots in Fig 7 show a statistical comparison between two- and three-segment procedures, and the box plots in Fig 8 show the comparison results between procedures accepted and rejected by the participants. In the graph, the cross indicates an outlier that is out of 99.3% coverage if the data are normally distributed. The plotted whisker extends to the minimum (or maximum) value that is not an outlier. The first and third quartile points are expressed using the box, and the median values are expressed using lines inside the box. One-way analysis of variance (ANOVA) was applied to test the obtained values of the shape indicators. Statistical analyses and graph creation were performed using MATLAB R2015b (MathWorks, Inc., Natick, USA).

**Table 2:** Obtained results of the three shape indicators for 10 CT datasets. *E*_*s*_ is the average shape distance between the native mandible and implanted fibular bones, *E*_*p*_ is the maximum projection, and *E*_*l*_ is the minimum length of the fibular segments.

	2segments			3segments		
ID	*E_s_*[mm]	*E_p_*[mm]	*E_l_*[mm]	*E_s_*[mm]	*E_p_*[mm]	*E_l_*[mm]
**1**	2.53±0.81	+6.19±1.60	51.90±9.19	1.33±0.23	+6.70±0.77	24.12±2.63
**2**	2.27±1.14	+4.47±1.23	38.17±9.65	1.39±0.28	+5.70±0.55	19.37±3.66
**3**	3.34±1.46	+6.00±1.80	54.48±12.09	1.68±0.58	+7.88±0.92	25.62±4.00
**4**	2.67±1.09	+5.69±1.68	47.57±9.40	1.63±0.57	+4.83±0.65	23.68±3.76
**5**	2.02±0.64	+7.08±0.95	48.78±11.33	1.51±0.24	+5.70±1.03	23.33±3.41
**6**	2.79±1.23	+5.05±1.47	50.57±10.49	1.61±0.34	+4.65±0.65	24.72±3.16
**7**	2.77±0.82	+5.73±0.90	44.55±9.82	1.61±0.30	+6.01±0.75	21.73±2.97
**8**	1.97±0.56	+5.70±1.66	42.45±11.13	1.60±0.36	+5.27±0.73	21.68±2.85
**9**	3.84±1.44	+7.78±2.54	54.43±9.73	1.93±0.39	+7.61±3.10	25.82±3.77
**10**	2.40±0.66	+6.39±1.94	50.22±8.72	1.26±0.26	+6.63±1.75	25.35±2.67

Fig 7A shows a significant difference in the shape distance *E*_*s*_ between the two- and three-segment procedures. This result indicates that three-segment reconstructions are more likely to achieve better reconstruction of the native mandible, which is mathematically reasonable (*i*.*e*., linear interpolation of the facial curved surface). Interestingly, however, a significant difference was not found in Fig 8A. The box plot shows that the participants rejected the outliers with a large shape distance and accepted moderate shape differences rather than choosing the minimum one. The rejected procedures with smaller shape distances were three-segment procedures; this means that two-segment procedures are relatively preferred for the given resection areas. No significant difference was found in the maximum projection *E*_*p*_ described in Figs. 7A and 8B. The graph shows that the participants protruded the fibular segments by an average of 6 mm from the original mandibular surface and accepted large variations for these cases. Fig 8C shows a significant difference in the minimum length of the fibular segments *E*_*s*_ between the accepted and rejected plans. Additionally, the data range provides valuable findings that the acceptable fibular segments are at least 20 mm long and that fibular segments more than 40 mm long are not used.

## Discussion

Preoperative planning has been treated as a case-by-case problem even in commercially available software solutions. The statistical analysis of planning data has potential benefit for systematic, quantitative understanding of the surgical procedures, but such usage of virtual planning has been rather unfocused. In this study, we have introduced three shape descriptors that quantify shape differences between pre- and post- mandibular shape from preoperative reconstruction plans. We focused on the use of multilateral indicators as an analysis tool for surgeons’ decision making in the literature of fibular transfer surgery. This retrospective study simulated surgical procedures using the developed software biGAKU and provides 120 planning results including the surgeon’s decision-making. The obtained data and the statistical analysis of the shape descriptors are thought to be useful as quantified medical knowledge regarding the number of fibular segments and placement of the connection points for patient-specific preoperative planning. The visual guidance provided by the shape distance map and the use of statistical data allow for a reduction in operating errors and the achievement of more reliable planning results, and would be commonly available for other interactive virtual planning software [7, 10]. Some planning results showed that the oral surgeons and dental technicians preferred a moderate shape distance rather than optimization of the shape distance to the smallest one. We discussed this finding with the participants, and they commented that a visually plausible shape can be empirically obtained when considering specific anatomical features such as the mental foramen and midline of the patient’s mandible. They considered the symmetric features of the human face in addition to local fitting of the fibular segments to the patient’s native mandible. Additionally, the surgical time required for the three-segment procedures was taken into account. These findings provide clear direction for further development of this planning system, and additional requirements for developing other shape descriptors.

There were some limitations to this study regarding the clinical applicability of the developed software. In this study, only the outer shape of the bony contour was considered to develop the shape descriptors. However, further clinical problems that influence the reconstruction should be considered, such as length of the vascular pedicle, size of the segments, location of the segments, and the oncological resection margins. In the current software, freely rotated virtual planes can provide unreal cutting edges. Due to the vascular supply of the fibular bone and the surrounding tissues, the cutting edges cannot be chosen without potentially compromising vascular supply to the bone. Introducing adequate physical restriction between virtual planes would solve this shortcoming and improve the reliability of the planning. The positioning of dental implants to allow functional reconstruction and rehabilitation of the chewing function should also be addressed. Recent studies have addressed major issues regarding clinical outcome and patient satisfaction; Olsson et al. [25] developed a haptics-assisted planning system that incorporates bone resection, fibular design, recipient vessel selection, pedicle and perforator location selection, and skin paddle configuration, and Zhao et al. [26] investigated the shape of the soft tissue flap being transplanted together with the bone flap. In contrast, the present study reported the use of biGAKU as a form of data science rather than concentrating on overall sufficiency as a clinical application, and quantified implicit surgical knowledge, especially the geometrical aspects of mandibular reconstructive surgery. To formulate the personalized reconstruction based on objective, quantitative indices will bring us closer to procedure standardization and automation of preoperative planning. Future research is warranted into clinical problems such as soft tissue reconstruction, length of the vascular pedicle, and prosthetic tooth replacement to restore chewing and biting function.

## Conclusion

This paper presents an improved design of preoperative planning with generalized quantitative shape indicators for fibular transfer in mandibular reconstruction and reports findings obtained through statistical analysis of the interactive planning results. The three shape descriptors are introduced as more generalized measures with which to evaluate the local difference between the reconstructed mandible and the patient’s original mandible. We focus on an asymmetrical, wide range of cutting areas including the mandibular sidepiece, and 120 planning results were obtained from a retrospective user study involving oral surgeons and dental technicians. For practical application of the obtained planning data, we can introduce some reasonable thresholds or constraints to the shape distance and maximum projection to interactive planning. Using the shape distance map for visual guidance will help with fine adjustment of the fibular segments. The proposed descriptors would also be useful as feature values to multilaterally evaluate reconstruction plans and systematically learn surgical procedures. Our future work will involve both extending the shape descriptors to further quantify implicit factors that affect the decision-making process in preoperative planning and investigating semi-automatic planning of mandibular reconstruction.

## Supporting Information

S1 Movie biGAKU demonstration movie. In response to the user’s operation in one virtual plane, othre virtual planes are updated simultaneously to maintain their connection and geometrical constraints. The local shape distance is spatially interpolated and mapped with the heat map color on the fibular segments.
(MPG)
